# Use of Post-Flotation Solidified Tailings from Copper Production for Ceramic Tile Production

**DOI:** 10.3390/ma16010132

**Published:** 2022-12-23

**Authors:** Piotr Izak, Yurii Delikhovskyi, Andrzej Olszyna

**Affiliations:** 1 Faculty of Materials and Ceramic, AGH University of Science and Technology, 30-059 Kraków, Poland; 2 Faculty of Materials Science and Engineering, Warsaw University of Technology, 00-661 Warszawa, Poland

**Keywords:** post-flotation sludge, tile ceramic, industrial waste disposal, utilization

## Abstract

The development of the mining industry has resulted in the accumulation of large amounts of waste, which effectively degrades the environment. The aim of this study is to check whether the addition of post-flotation sludge from copper production to the typical ceramic mass of the “*gress porcelanato*” will allow to obtain qualified sintered or faience tiles. By adding successive amounts of post-flotation sludge to the high-quality lamellar mass, typical parameters such as firing shrinkage, water absorbability and bending strength after firing were assessed. The structure of the obtained ceramic materials, using scanning electron microscopy (SEM with EDS), was also determined. Obtaining positive results would allow not only to significantly reduce the production costs of ceramic tiles, because the sludge is finely divided and no grinding is necessary, but, above all, will allow to eliminate the environmental risk. The present study has shown that it is possible to introduce up to 20% post-flotation sludge for *gres porcellanato* tile production and up to 50% post-flotation slugde for faience tile production. Both types of ceramic materials with an appropriate proportion of sludge, meet the requirements of tile standards in terms of mechanical strength and water absorption.

## 1. Characteristics of Fine Ceramics

Generally, ceramic materials, due to their water absorbability, can be divided into: porous and sintered [1]. The basic ingredients for the production of fine ceramics are quartz, feldspar (potassium, sodium) and a clay substance consisting of clays (clays and kaolins) [2,3]. Depending on the type of fine ceramics, raw materials may vary significantly. In particular, this applies to ceramic tiles [4,5].

**Figure 1:**
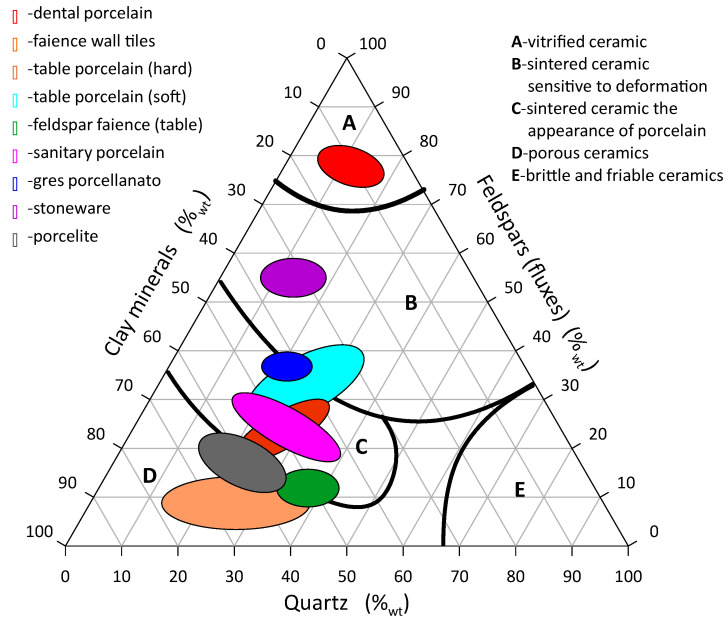
Diagram of typical rational compositions of ceramic masses.

Describing the raw material compositions of these materials by means of the mentioned components, the areas of rational compositions of typical masses of ceramic materials are shown in the Figure 1.

Although the raw materials from different deposits vary in their mineral and chemical compositions, the so-called rational composition is very useful in production practice as it allows to obtain a material with desired parameters.

**Figure 2:**
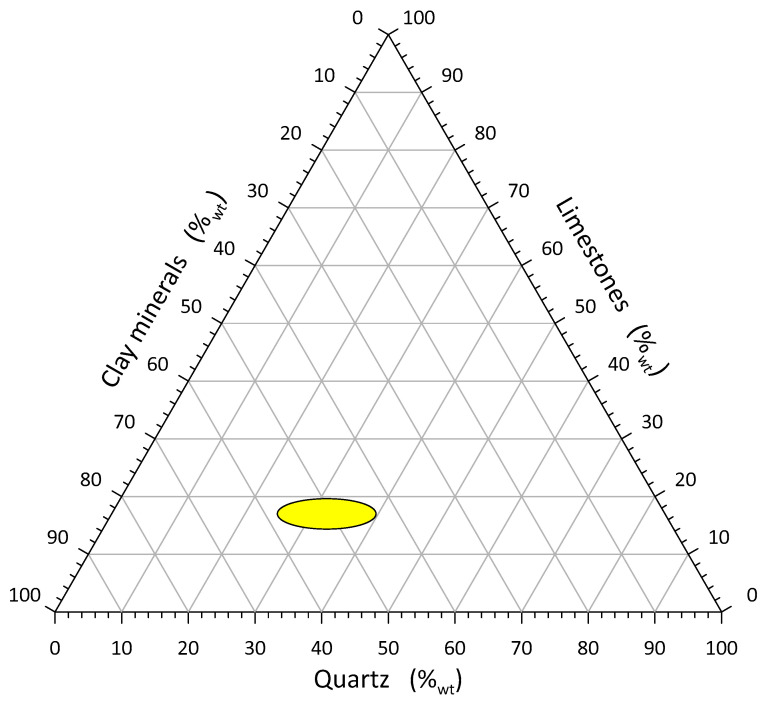
Diagram of typical rational compositions of ceramic masses with limestone’s.

Among the mentioned, there is also a ceramic material containing limestone, called lime- or feldspar-faience [1]. Feldspar faience contains in its composition sodium-calcium feldspars. Calcium faience, on the other hand, has a high content of calcium carbonate (Figure 2). Calcium carbonate can be added in the form of chalk, marble or marl and constitute an additional group in the rational composition. It can also be added simultaneously with CaCO_3_ and MgCO_3_ in the form of dolomite. These types of faience are fired at a temperature of 1000–1180 °C. In particular, calcium faience has a light body with a low apparent density and water absorbability. The porosity and brittleness of the body increases with increasing calcium content.

The average water absorbability of lime faience is 20% by mass, although for wall tiles, it does not exceed 18%. Since the faience body is permeable to water and gases, it absorbs liquids and gets dirty quickly, which is why it is usually covered with glaze.

In the case of the production of semi-porcelain ceramic tiles, also called as *vitrified* tiles, utility properties intermediate between stoneware and porcelain can be obtained. Its body is non-translucent, sintered and characterized by water absorbability below 6% and high mechanical strength [2]. Semi-porcelain is a cheaper material than porcelain, because its production uses lower-quality raw materials, but, on other hand, is much more expensive than faience due to the higher firing temperature.

A special type of ceramic tile is the so-called *gres porcellanato*. In this case, the water absorption rate must not exceed 0.5%_wt_ and the mechanical flexural strength be less than 35 MPa [3,4,5]. General rational composition is around 30%_wt_ fieldspar, 25%_wt_ quatrz and 45%_wt_ clay minerals (see Figure 1). Since this type of ceramic is the most popular, it was used for comparison purposes. *Gres porcellanato* (Nowa Gala) production mass was used as a base for the study.

## 2. Characteristics of Tailings Slurry

Generally, post-production wastes are threatening to all components of the environment, earth’s surface, the hydrosphere, the biosphere and the atmosphere. These threats basically occur at all stages of waste management, i.e., during their production and collection, transport, utilization and disposal, as well as during their storage [6].

Flotation tailings are the dominant type of waste generated in the copper ore enrichment process and account for about 94% of the mass of produced wastes. It is a mineral waste in the form of a finely graded gangue, which is essentially neutral to the environment and harmless to health. In the case of Poland, this waste is produced in the amount of about 24 million Mg/year and is landfilled. Until now, over 800 million Mg of this waste has been deposited in the copper industry’s landfills and, in principle, it is utilized in a minimal extent [7]. Attempts to use flotation waste concerned the so-called *mining concretes*, i.e., mixing with ashes from coal combustion, and as a sealing medium for the production of *self-solidifying* mineral powder, as well as in terms of *neutralization* of sulfuric acid. Overall, about 1% of flotation wastes is utilized, the remainder is the so-called anthropogenic deposit–resource reserve of trace elements [8]. Tailings from copper production both solidified and in the form of slurries have so far not been used for tile production.

The particle size composition of waste produced from sandstone ore is dominated by the grain fraction below 0.06 mm, whose share ranges from 62 to 66%, while the amount of the coarser grain fraction above 0.2 mm is approx. 8%. The waste mainly consists of inert silica, aluminosilicates and carbonate minerals [9].

The chemical and mineralogical composition of post-flotation waste is variable and depends on the type of processed ore. In the waste generated as a result of the sandstone ore flotation process, the predominating part takes quartz, approximately 40%, and carbonate minerals in face of dolomite—30%, and calcite,10%; those are the most common wastes. Calculated as oxides, those are approximately 50% SiO_2_, 13% CaO and 5% MgO, while fine-grained carbonate waste contains about 24% CaO, 20% SiO_2_ and more than 5% MgO [10,11]. This also applies to other slurries usually burned through (sewage), mostly for the production of bricks [12].

Carbonate waste is finer-grained, and the share of the mentioned grain fractions are 90% and approx. 3%, respectively. Taking into account the good fineness of the waste, it is hereby proposed to use them also for the production of ceramic tiles.

For sintered ceramic tiles, the temperature and width of the sintering interval play an important role, especially contain lithium silicate [13] in composition with other fluxes [14,15].

## 3. Materials and Methods

The tests were carried out on 8 sets of masses containing a different amount of post-flotation sludge and *gress porcelanato* lamellar mass (Nowa Gala) (Table 1). All masses were prepared and tested in the same way. The compositions of each set are given as weight percentages.

The following scope of research was assumed:

- Firing of formed beams in a gradient furnace in the temperature range 800–1250 °C (KENT);
- Determination of the mechanical bending strength after firing (Wick Roell Z150);
- Determination of post-burn shrinkage;
- Testing water absorbability;
- Examination of the microstructure on a scanning microscope SEM (JEOL–5400) with additional Energy-dispersive X-ray spectroscopy EDS;
- Particle size distribution; Mastersizer 2000 (Malvern);
- Table 1 shows the chemical analyzes (converted into oxides) of the raw materials. The underlined oxides were used to calculate rational compositions (Table 2).

**Figure 3:**
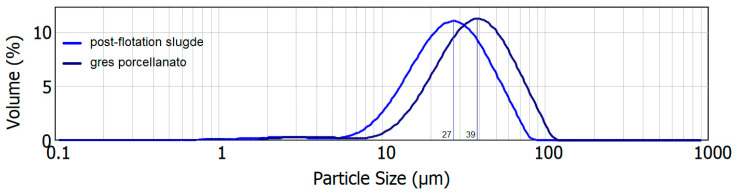
Grain distributions of sludge and *gres porcelanato* NG raw materials.

The grain distribution of *gres porcelanato* NG and sludge is shown in Figure 3.

The shapes of the curves represent similar unimodal distributions. The most common grain fraction in *gres porcelanato* NG slurry relative to sludge is about 12 μm larger. The successive introduction of the typical *gres porcellanato* mass into the flotation sludge increases the amount of clay minerals by approx. 11%, quartz by approx. 14%, feldspar by approx. 12% and decreases the proportion of carbonate minerals by approx. 38%. (Na + K)-feldspar act as low–temperature fluxes [5]. Such a change in the rational composition results a significant change in the properties of the ceramic material after firing, i.e., from porous ceramics to sintered ceramics, taking into account different firing temperatures. The research was treated as reconnaissance. Comparing the rational compositions with Figure 1, it is clear that the field of compositions includes porous ceramics D and sintered ceramics C, i.e., faience and semi-porcelain. Comparing the compositions with Figure 2, the mixtures fall within a far more extended range of calcium faience than the literature information predicts.

## 4. Properties of Ceramics with Sludge

Samples in the form of compressed beams dimensions 80 × 9 × 10 mm^3^ were used for the tests. Seven samples were made from each set. They were dried and fired in a gradient furnace in the temperature range of 900–1250 °C in two rounds.

### 4.1. Subsection

**Figure 4:**
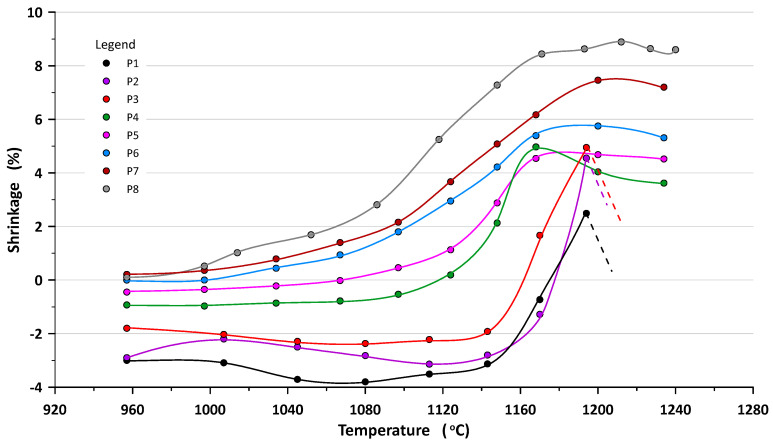
Shrinkage as a function of firing temperature.

The results of the firing shrinkage tests as an average value of 7 samples are shown in Figure 4. The standard deviation of each measurement point did not exceed 5% but for samples containing 50% sludge and fired above 1160 °C was more than 15%. The research showed that the increasing content of the sludge in the mass results in decreasing of the shrinkage rate of the material in the entire temperature range. Moreover, even with the sludge content above 20% there is a noticeable expansion of the ceramic material up to the temperature of 1100 °C, which in the temperature range 1150–1180 °C quickly decreases so that the samples P1–P3 above 1180 °C were melting. In the case of pure sludge, the expansion at 1080 °C reaches 4%. This proves that the sludge has a high carbonate content. The sample containing the mass of *gres porcellanato* shrinks successively during sintering and stabilizes at the level of 8.5% in the temperature range 1170–1240 °C.

### 4.2. Flexural Strenght

**Figure 5:**
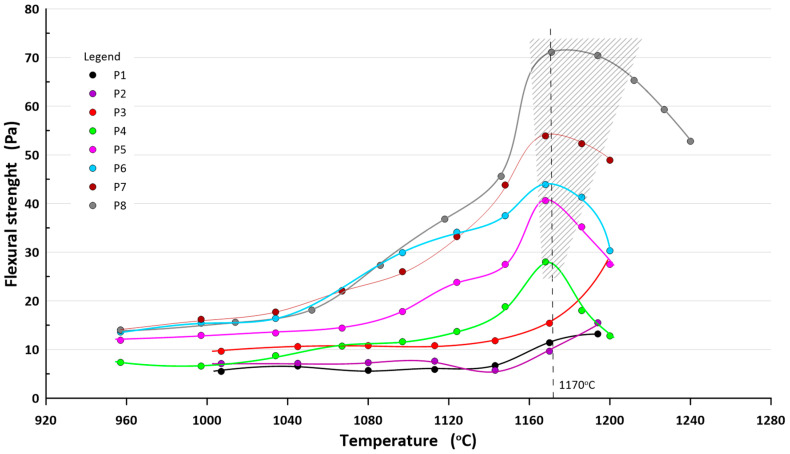
Mechanical bending strength as a function of firing temperature.

The mechanical bending strength after firing the masses P1–P8 is shown in Figure 5. The results at each temperature are the average value of 7 samples. The standard deviation of each measurement point did not exceed 6%, but for samples containing 50% sludge and fired above 1160 °C was more than 10%.

First of all, it should be noted that the highest mechanical strength is achieved by samples fired at a temperature of approx. 1170 °C. The strength decreases rapidly with increasing sludge content in the bulk.

The highest mechanical bending strength (>30 MPa) was obtained for the P5, P6, P7, P8 compositions fired at a temperature of about 1170 °C. The lowest mechanical strength is demonstrated by samples contained more than 40% of sludge. In this case, the mechanical bending strength does not exceed 28 MPa.

### 4.3. Water Absorbability

**Figure 6:**
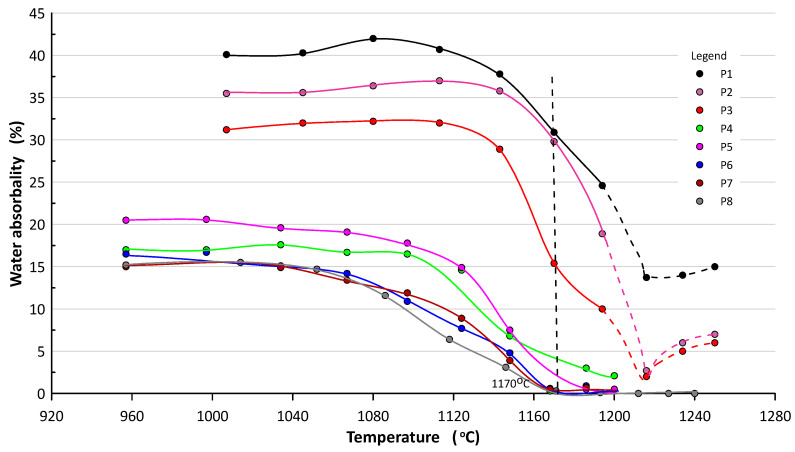
Changes in the water absorbability of ceramics as a function of firing temperature.

The results of water absorbability measurements for individual samples after firing are presented in Figure 6. The results in weight percent are average value of 14 samples. The standard deviation of each measurement point did not exceed 4.5%, except for samples fired above 1200 °C. In this case, the samples contained a lot of closed and open pores and accurate measurement was difficult.

The tests showed that the water absorbability of the tested materials after firing depends on both the firing temperature and the sludge content. As the firing temperature is lowered, there is a marked increase in absorbability for compositions containing less than 70% sludge. From a temperature of about 1170 °C for P6–P8 sets, the absorbability value is close to zero. P5–P8 masses show the best sintering tendency. With an increased share of sludge at temperatures above 1170 °C, the water absorption initially decreases and then increases (dashed line in chart), which is the result of melting and expansion of the material. Lightweight expanded clay aggregate (LECA) is formed.

### 4.4. Structure of Ceramics

**Figure 7:**
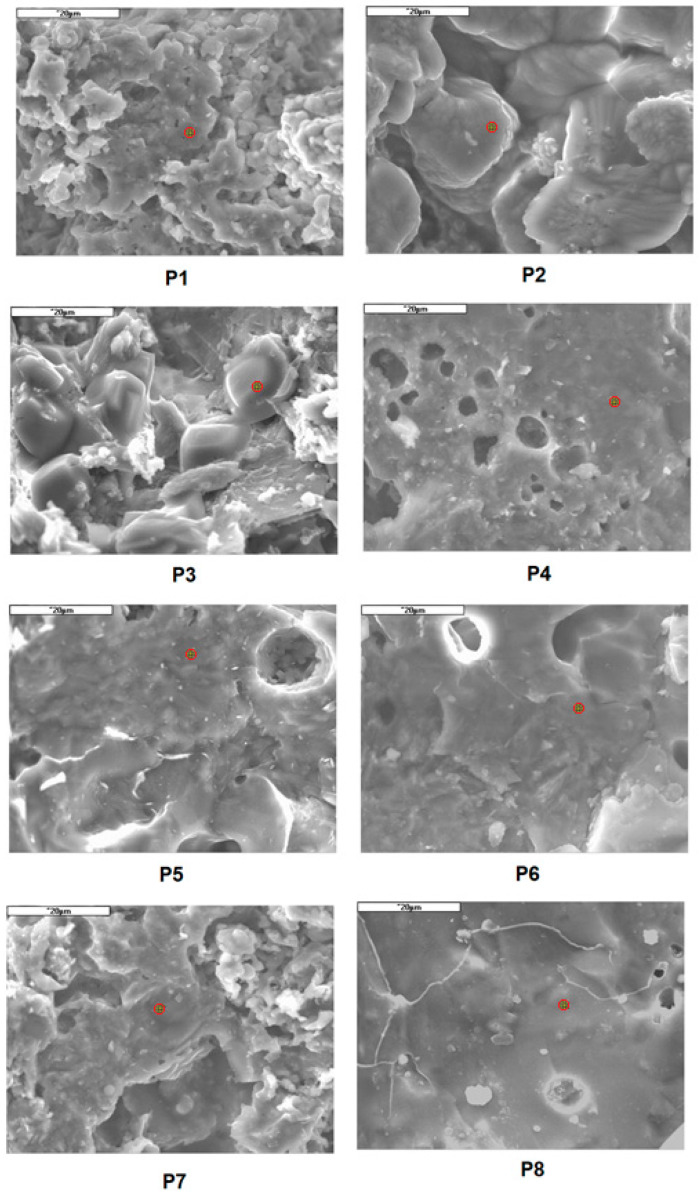
Microphotographs of samples containing a successively increased amount of *gres porcellanato* mass.

**Figure 8:**
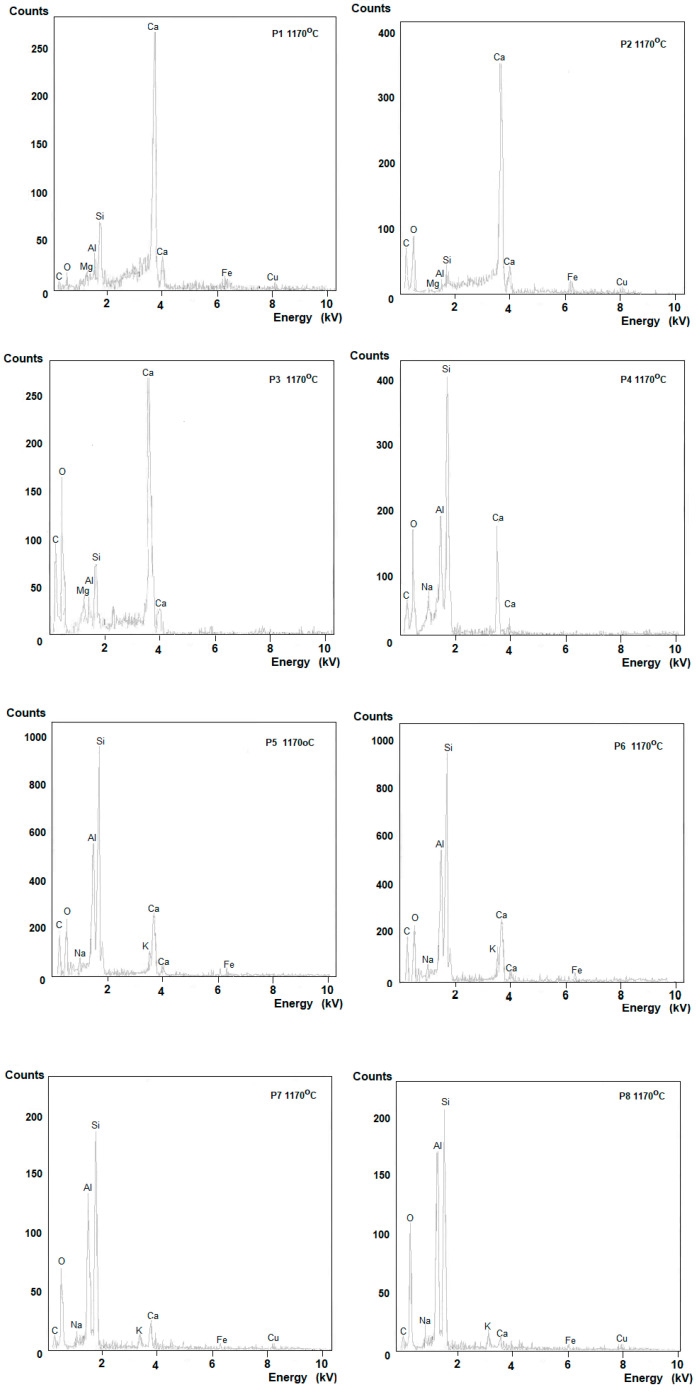
EDS analysis of the tested samples at the points marked in Figure 6.

Samples fired at a temperature of 1170 °C, i.e., the ones with the highest mechanical bending strength, were selected for microscopic examinations and EDS. Depending on the sludge content, the structure of ceramic materials differs significantly (Figure 7 and Figure 8).

All the tested samples, except P8 and P7, are characterized by a significant presence of pores. At the temperature of 1170 °C, the P1–P3 compositions contain weakly bound crystals which firstly grows and then diminishes as the content of *gres porcellanato* mass increases. The liquid phase is already visible in samples P4–P8.

Averaged EDS analysis for samples P7 and P8 showed the presence of Al probably related to the presence of mullite, although no needles of secondary mullite were noticed. On the other hand, in the P1–P3 masses, a low quartz content and an exceptionally high presence of Ca can be observed. Scanning microscope images of the samples show that the masses containing increased amounts of sludge have more pores, which may be caused by thermal dissociation of mainly calcium carbonate during firing.

## 5. Discussion

The preliminary tests carried out on eight masses containing various amounts of flotation sludge and *gress porcelanato* mass should be summarized in two areas: possibilities of using the sludge for the production of absorbent faience-type tiles and maximum amount of sludge additive to the sintered masses in order to reduce the firing temperature.

Research shows that shrinkage increases as the firing temperature increases, which indicates sintering of the masses. The sludge increases the sintering ability of masses and narrows the sintering interval. This fact is confirmed by water absorption measurements. The more sludge by weight added (>40% CaCO_3_), the higher the water absorbability values and the narrower the sintering process is, especially at temperatures above 1150 °C.

Masses with low sludge content (<30%) start sintering at lower temperatures, i.e., from 1150 °C. At a temperature of about 1170 °C, P1–P4 compositions have zero water absorption.

The analysis of the results of mechanical bending strength shows that the masses fired at a temperature of approx. 1170 °C have the highest strength values and it decreases with increasing sludge content.

**Figure 9:**
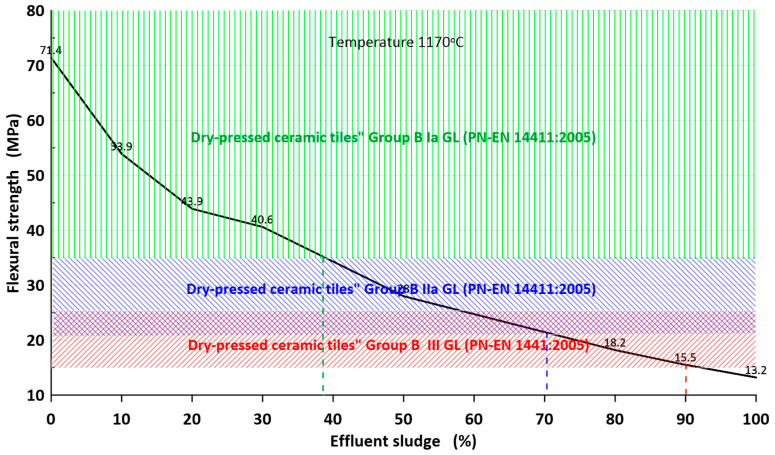
The influence of the sludge content in the tiles mass on the mechanical bending strength in terms of the requirements for wall and floor ceramic tiles.

From the point of view of the parameters of ceramic tiles according to PN-EN 14411: 2005, most sets (except for pure sludge) are allowed for production. This applies to faience wall tiles (B III GL) and non-frost-resistant tiles (B IIa) as well as frost-resistant *gres porcellanato* tiles (B Ia) (Figure 9).

In order to meet the standard requirements of frost-resistant ceramic sintered *gres porcellanato* tiles, a maximum of 40% of sludge can be included. However, taking into account the width of the sintering interval, the addition of the sludge should not exceed 20% (Figure 9).

It should be noted that with a higher content of sludge in the composition, i.e., (from 40 to 80%), only porous material can be produced, e.g., glazed faience tiles or stoneware tiles. This fact is confirmed by textural (microscopic) tests. A further increase in the amount of sludge results in more pores what is mainly related to the decomposition of calcium carbonate.

EDS tests confirmed the presence of calcium, silicon, aluminum and magnesium atoms. At temperatures above 1050 °C, it suggests the formation of solid solutions, probably calcium (and magnesium) aluminosilicates.

A thorough analysis in this area requires detailed X-ray examinations, especially since in these systems with the participation of alkali, many silicate and aluminosilicate compounds may be formed [16].

**Figure 10:**
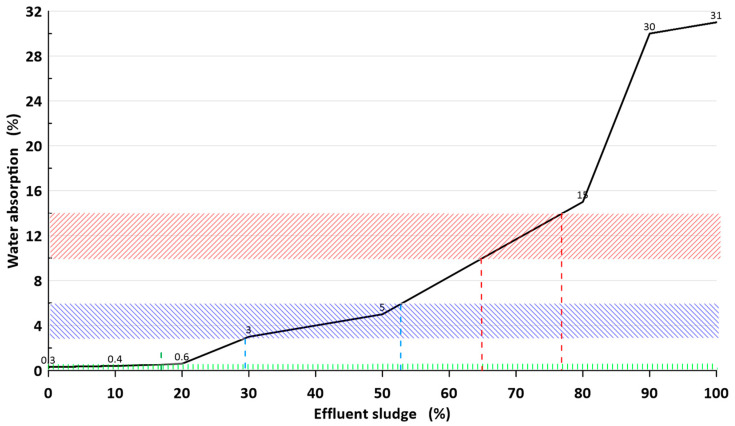
Influence of the sludge content in the lamellar mass on water absorption. Designation of area ranges as in Figure 8.

Much more restrictive parameters of ceramic tiles concern water absorption (Figure 10).

In this case, frost-resistant tiles of group B Ia (*gres porcellanato*) should contain less than 18% of sludge. Group B IIa frost-resistant floor tiles may contain 29–53% of sludge. And group B III wall tiles may contain 65–77% of sludge [17]. This is interesting because usually sludge is used to make ceramic bricks [18,19,20,21,22,23,24,25].

### 5.1. Conclusions

Based on the current study, the following conclusions can be drawn:

1. The compositions of the typical *gres porcellanato* mass and flotation sludge (Rudna) allow for the production of three types of ceramic tile materials at a firing temperature of approx. 1170 °C;
2. Flotation sludge generally reduces the mechanical strength of the material and increases its water absorbability;
3. With a sludge content of up to 18% in the tile base mass, a frost-resistant sintered *gres porcellanato* porcelain is obtained, with a mechanical bending strength of approx. 35 MPa and a water absorption capacity of less than 0.5%;
4. With a sludge content of 28–53%, a non-frost-resistant (stoneware) plate material is obtained with mechanical bending strength of 21–35 MPa and water absorption within 3–6% by weight;
5. With a sludge content of 65–77%, a faience-type plate material with mechanical bending strength of 18–25 MPa and water absorption within 10–15% by weight where obtained.
6. The post-flotation sludge contains large amounts of calcium carbonate and/or dolomite, which significantly affects the sintering interval and burning temperature of the material as well as other operational and technological parameters.

### 5.2. Future Directions and Limitations

The tailings sludge from copper production can contain varying amounts of water. For this reason, it can be difficult to incorporate it into the ceramic mass. Further research should take this aspect into consideration. Due to the significant changes in the parameters for the tested sets fired at temperatures above 1100 °C, especially those containing larger amounts of tailings sludge, follow-up measurements should also be made with special attention to measurement errors.

Also of interest may be mixtures of raw materials of various mining wastes together.

## Figures and Tables

**Table 1:** Chemical analysis of raw materials.

	Oxides	Gres Porcellanato NG [%_wt_]	Sludge [%_wt_]
1	SiO_2_	74.4	35.4
2	Al_2_O_3_	16.0	12.4
3	CaO	0.0	28.8
4	MgO	0.1	5.4
5	K_2_O	1.2	2.8
6	Na_2_O	3.8	0.2
7	Fe_2_O_3_	0.4	2.6
8	MnO	0.1	0.2
9	TiO_2_	0.2	0.2
10	LOI	3.2	11.5
11	H_2_O	0.6	0.5
Sum		100	100

**Table 2:** Rational composition of sets.

Mass Symbol	Gres Porcellanato NG [%_wt_]	Sludge [%_wt_]	Clay Minerals [%_wt_]	Quartz [%_wt_]	(Na + K)-Feldspar [%_wt_]	Carbonates [%_wt_]
P1	0	100	19.9	11.0	15.4	53.7
P2	10	90	21.7	13.0	17.2	48.4
P3	20	80	23.2	15.0	18.9	42.9
P4	50	50	28.1	20.8	24.2	26.9
P5	70	30	31.5	24.7	27.7	16.1
P6	80	20	33.2	26.7	29.4	10.7
P7	90	10	34.8	28.6	31.2	5.4
P8	100	0	36.5	30.6	32.9	0

## Data Availability

Not applicable.
